# IL28A protein homotetramer structure is required for autolysosomal degradation of HCV-NS5A in vitro

**DOI:** 10.1038/s41419-020-2400-9

**Published:** 2020-03-23

**Authors:** Yuan-yuan Ma, Jian-rui Li, Zong-gen Peng, Jing-pu Zhang

**Affiliations:** Key Laboratory of Biotechnology of Antibiotics, the National Health Commission (NHC), Beijing Key Laboratory of Antimicrobial Agents, Institute of Medicinal Biotechnology, Chinese Academy of Medical Sciences and Peking Union Medical College, Beijing, 100050 China

**Keywords:** Macroautophagy, Molecular biology

## Abstract

Interferon lambda-2 (IL28A) has a wide antiviral effect with fewer side-effects. Autophagy is a host mechanism to maintain intracellular homeostasis and defends invasion of pathogenic microorganisms. HCV NS5A can disable host defense systems to support HCV replication. Thus, molecular mechanism of interaction among interferon lambda, autophagy, and HCV was concerned and explored in this study. We report that HCV NS5A activated an incomplete autophagy by promoting the autophagic ubiquitylation-like enzymes ATG3, ATG5, ATG7, ATG10, and autophagosome maker LC3B, but blocked autophagy flux; IL28A bound to NS5A at NS5A-ISDR region, and degraded HCV-NS5A by promoting autolysosome formations in HepG2 cells. A software prediction of IL28A protein conformation indicated a potential structure of IL28A homotetramer; the first α-helix of IL28A locates in the interfaces among the four IL28A chains to maintain IL28A homotetrameric conformation. Co-IP and cell immunofluorescence experiments with sequential deletion mutants demonstrate that IL28A preferred a homotetramer conformation to a monomer in the cells; the IL28A homotetramer is positively correlated with autolysosomal degradation of HCV NS5A and the other HCV proteins. Summarily, the first α-helix of IL28A protein is the key domain for maintaining IL28A homotetramer which is required for promoting formation of autolysosomes and degradation of HCV proteins in vitro.

## Introduction

Hepatitis C virus (HCV) is an important human pathogen. HCV infection incidence has increased globally and its complication remains a problem for a long time. In recent years, several medicines directed against HCV proteins (direct-acting antiviral agents, DAAs) have been in clinical use, including NS3/4A-targeted Telaprevir and Simeprevir, NS5A-targeted Daclatasvir and Ledipasvir, and NS5B-targeted Sofosbuvir, which have greatly improved treatment of HCV infection. Unfortunately, a number of reports of drug resistance have emerged as more and more patients being treated with DAAs^1–5^. Thus, host factors with anti-virus property have been got attention again. Interferons (IFNs) are the first line of host defense against invading viral pathogens^6^. IFNs are classified as type I, type II, and type III based on their structural features, receptor usage, and biological activities^7^. For decades, type I IFNs have been considered indispensable and important antiviral mediators. Interferon-alpha (IFN-α) plus ribavirin (RBV) combination antiviral therapy has been used as the standard treatment for patients with chronic HCV infection for more than 10 years, but the HCV clear efficiency is only in 50% of patients^8^. Type II IFN is limited in direct antiviral effect, but have pleiotropic effects on a diverse set of immune cells promoting both adaptive and innate responses^9^. Type III interferons, also called Interferon λs (Interferon λ1–IL29, Interferon λ2–IL28A, Interferon λ3–IL28B), as a new class of IFNs which share several common features with type I IFNs, have functions in antiviral, antiproliferative, and antitumor activity, but with fewer side-effects^10^. Exogenously administered IFN-λs have been shown to inhibit the replication of a wide variety of viruses, such as VSV, EMCV, HBV, HCV and human cytomegaloviruses in vitro and vaccinia virus and herpes simplex virus type 2 in vivo^11,12^. However, whether IFN-λs directly target and degrade virus components is unclear. Thus, IL28A was studied in this work as a representative member of the type III interferon family.

Autophagy is one of the homeostatic mechanisms the host cell employs to clear unused intracellular materials and impaired organelles through lysosomal degradation^13,14^. Moreover, many studies have reported that autophagy has a synergistic action with interferons in defending the host from many pathogen infections^15–22^. Autophagy dysfunction is usually associated with various pathologies, including cancer, infectious diseases, and neurodegenerative disorders^23–26^. However, some studies present contradictory opinions respecting autophagy facilitating HCV replication, namely, how HCV exploits the autophagy pathway to support its replication. Wang and Ou reviewed the impacts of HCV proteins on autophagy pathway, such as HCV NS3/4A protease binding to mitochondria-associated IRGM, HCV NS4B forming a complex with Rab5, hVps34 and Beclin-1, and HCV core activating UPR, HCV NS5A upregulating the expression of Beclin-1, and HCV NS5B binding to ATG5, and so on; all the actions of the HCV proteins benefit to promote autophagosome formation to support virus replication^27^. The autophagy pathway activated by viruses is usually incomplete, which cannot cause elimination of the viruses due to both inhibition in autolysosome formation or in lysosomal degradation^28–32^. HCV NS5A is a nonstructural (NS) protein critical for HCV RNA replication, virion assembly, and interactions with cellular immunity factors^33–35^ because it directs the virus replication complex to translocate to the ER and autophagosome membranes^36,37^. The ISDR sequence of NS5A is a critical domain for NS5A interaction with IFN-alpha in clinic treatment response^38^. However, until now, there have been no reports describing the direct interactions among the HCV NS5A protein, IFNλs, and autophagy. Our previous studies showed that the IL28A protein cooperated with ATG10S to degrade HCV sub-replicons by promoting fusion of autophagosome to lysosomes^39,40^. In this study, we explored whether IL28A has direct interactions with HCV NS5A protein and the autophagic apparatus, and how IL28A plays its roles in promotion of complete autophagy pathway to eliminate HCV NS5A and other HCV proteins. We find that the HCV proteins can be cleared by IL28A homotetramer mediated complete autophagy process. We hope that the findings will make a substantial contribution to the understanding of IL28A anti-HCV mechanism and will provide new clues for the development of anti-HCV medicines.

## Results

### The HCV NS5A protein induces incomplete autophagy in HepG2 cells

**Fig. 1:**
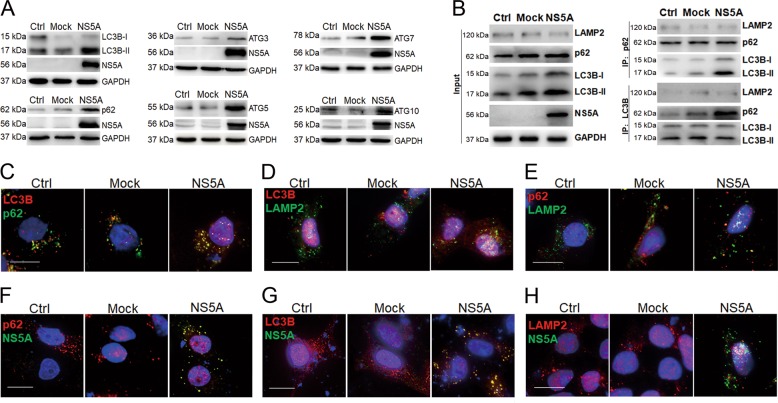
Incomplete autophagy induced by the HCV NS5A protein in HepG2 cells. HepG2 cells were transfected with pIRES2-NS5A expression plasmid (NS5A group), or with the empty vector (pIRES2-EGFP plasmid) transfection as a mock group (Mock), and non-transfected treatment as a control group (Ctrl), and cultured for 48 h. **a** Western blotting showed that HCV NS5A overexpression elevated levels of LC3B, p62, ATG3, ATG5, ATG7, and ATG10 proteins compared to the two control groups. A histogram of the relative levels of ATG3, ATG5, ATG7, ATG10, LC3B-II/I, and p62 proteins are shown in Fig. S1A. **b** Co-IP results showed interaction between LC3B with p62, but without LAMP2 induced by HCV NS5A. **c**–**h** Cell Immunofluorescence experiments for detecting the effects of HCV NS5A on the subcellular co-localization among LC3B, p62, LAMP2, and HCV NS5A. The results indicated that NS5A promoted co-localization of LC3B with p62 (**c**) but not of LAMP2 with LC3B (**d**) and with p62 (**e**). Also, co-localizations of NS5A with p62 (**f**) and with LC3B (**g**), but not with LAMP2 (**h**) were observed. Scale bars = 15 μm. The scatter diagrams for co-localized particles per cell, and their Pearson coefficients can be seen in Figs. S1C–G.

HCV can interfere with host defense mechanisms including immunity and autophagy by HCV proteins interacting with host factors. Respecting that HCV NS5A has a critical role in HCV genome replication through docking the HCV replication complex to the autophagosome membrane^36,37^, we examined whether the HCV NS5A protein functions in formation of autophagosomes. An HCV NS5A expression plasmid was constructed and transfected into HepG2 cells, and autophagy marker, ratio of LC3B-II to LC3B-I, and the selective receptor p62 protein were estimated by western blot. The result showed the levels of LC3B-II/I and p62 protein significantly higher in the NS5A-expressing cells than in controls (Fig. 1a), which implies that NS5A activated autophagy. We also tested autophagy-related proteins and heterodimer that are involved in the two autophagy ubiquitination systems: ATG3, ATG5-ATG12, ATG7, and ATG10; HCV NS5A protein also induced higher expression of the four proteins than the mock group (Fig. 1a). NS5A promoted combination of LC3B with p62 as confirmed by Co-IP tests; however, it did not promote interactions between LC3B and the lysosomal membrane protein LAMP2, and between p62 and LAMP2 (Fig. 1b). Further, Cell immunofluorescence assay showed that significantly more p62 co-localized with LC3B in cells with the HCV NS5A expression than in control HepG2 cells or in the mock-transfected groups (Fig. 1c). However, NS5A expression did not promote the co-localization of LC3B with LAMP2 (Fig. 1d) or p62 with LAMP2 (Fig. 1e). Moreover, the HCV-NS5A protein co-localized with p62 (Fig. 1f) and with LC3B (Fig. 1g) confirm that NS5A was bound to both the selective receptor p62 and autophagosomes. However, NS5A did not interact with LAMP2 (Fig. 1h). These results suggested that NS5A induced an incomplete autophagy process, leading to the accumulation of autophagosomes, and blocked the fusion of lysosomes with autophagosomes; in other words, HCV-NS5A expression provided a larger area of membrane surface for HCV replication but inhibited lysosomal degradation.

### IL28A promotes degradation of NS5A by inducing the formation of autolysosomes

**Fig. 2:**
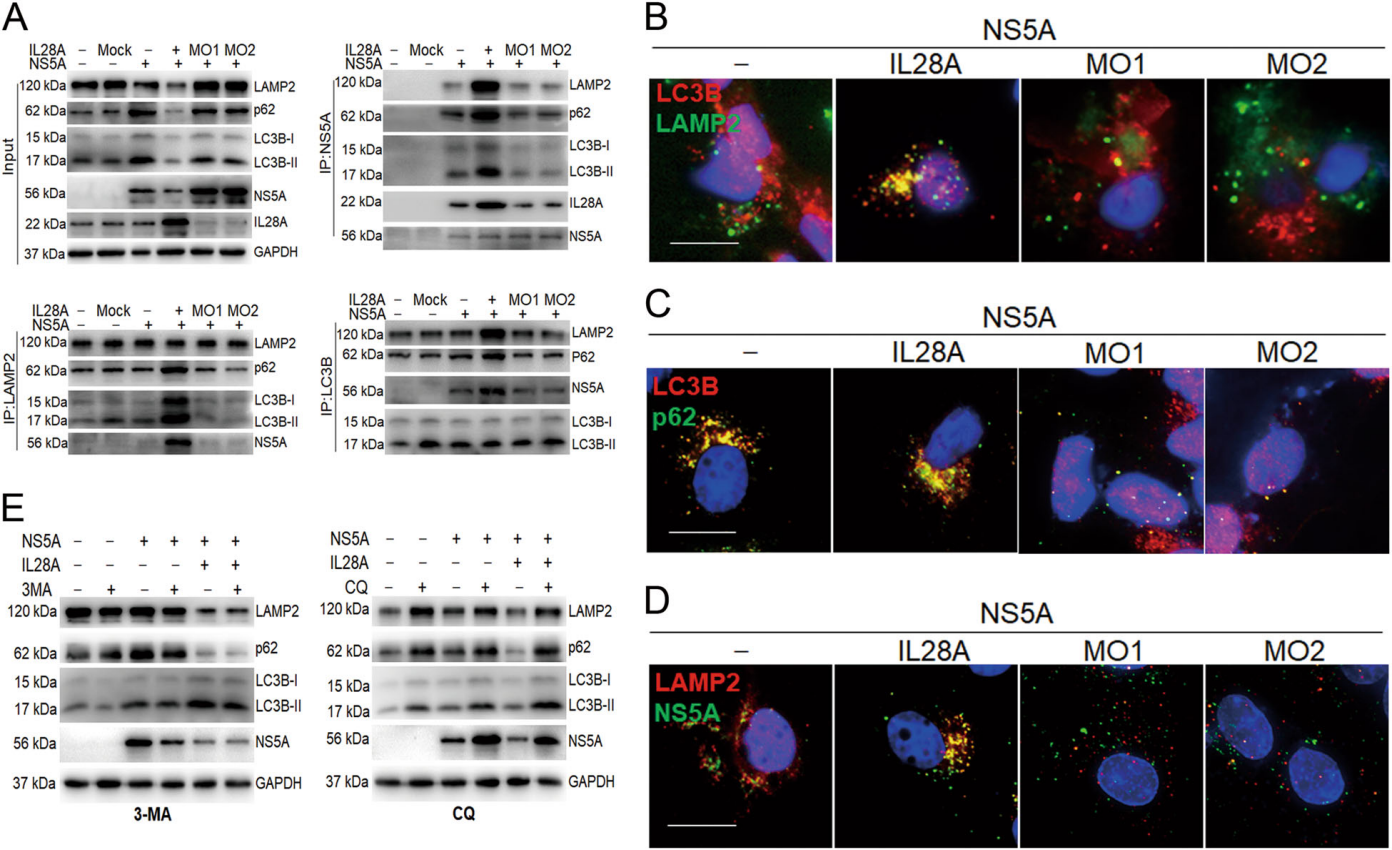
Degradation of the HCV NS5A protein enhanced by IL28A through promotion of autolysosome formation. **a** Co-immunoprecipitation was used to detect IL28A effect in decrease of HCV NS5A, the formation of autolysosomes and autophagy activity via upregulation and downregulation IL28A expression. pIRES2-EGFP (as the mock), IL28A overexpression construct, and *il28a*-MO1 and *il28a*-MO2 were separately transfected into HCV-NS5A expressing HepG2 cells. Non-transfection and only mock in HepG2 cells were as the two control groups. **b**–**d** Cell Immunofluorescence experiments were used to determine correlation of IL28A level with the subcellular co-localization of LC3B-LAMP2 (**b**), LC3B-p62 (**c**) and NS5A-LAMP2 (**d**) in the HCV-NS5A expressing HepG2 cells under the same cell treatments as in (**a**). The minus (–) indicates the HCV-NS5A expressing HepG2 cells without treatments by IL28A up- and downregulation. The MO1 and MO2 are two IL28A antisense morpholino oligomers that can downregulate endogenous IL28A expression in the target cells. All the scale bars are 15 μm. The scatter diagrams of particles containing co-localized LC3B-LAMP2, LC3B-p62, and NS5A-LAMP2, and their Pearson coefficients can be seen in Fig. S2. **e** Autophagy inhibitors 3-MA and CQ were used to detect the phase of IL28A action in the autophagy process through checking the marker proteins of autphagy flux in the NS5A-HepG2 cells by western blotting test. The NS5A-HepG2 cells were transfected with IL28A expression construct compared with no IL28A construct, and exposed to 3-MA- or CQ-containing medium.

Our earlier studies showed that IL28A activated and promoted a complete autophagy process in which the HCV sub-genomic replicon was inhibited^39^. In this study, we asked whether IL28A could also degrade the NS5A protein by promoting autophagy flux. Co-immunoprecipitation tests showed that IL28A overexpression was associated with decreased levels of NS5A, LAMP2, p62, and LC3B proteins (Fig. 2a), indicating that autophagy flux was smooth and the NS5A protein had been degraded compared to transfection with NS5A alone. In contrast, knockdown of endogenous IL28A expression by MO*il28a* reversed the results—the NS5A protein level was much higher than in the group of NS5A alone, and the p62, LC3B, and LAMP2 levels recovered to the levels of the NS5A group (Fig. 2a). These results indicate that IL28A plays a role in the degradation of NS5A protein. Meanwhile, Co-IP results showed that IL28A overexpression promoted interactions among LAMP2, LC3B, p62, IL28A, and NS5A proteins, which implies the formation of autophagolysosomes containing NS5A-p62 complexes; conversely, IL28A knockdown significantly reduced the association among these proteins (Fig. 2a). Cell Immunofluorescence double staining experiments confirmed that IL28A overexpression led to the formation of the complexes containing LAMP2 associated with LC3B and with NS5A, together with LC3B-p62 aggregates, compared to the NS5A group. Conversely, the colocalized particles of LAMP2 with LC3B, LAMP2 with NS5A, and LC3B with p62 were almost absent in cells of *il28a* knockdown groups with MO*il28a* transfection (Fig. 2b–d). These results demonstrated that IL28A facilitated the formation of autolysosomes and normal autophagy flux that led to the breakdown of the NS5A protein. However, at which stage of autophagy process IL28A exerts its action is not known. We used two autophagy inhibitors [3-methyladenine (3-MA) and chloroquine (CQ)] to study IL28A effects on NS5A levels and autophagy flux. We found that CQ blocked autophagy flux and increased NS5A level no matter whether IL28A was overexpressed compared with the results of NS5A and IL28A were co-expressed. These results suggested that IL28A may act before lysosomal degradation because CQ functions to increase the pH and inhibit the digestive activity of lysosomes (Fig. 2e). The inhibitor 3-MA that interferes with the formation of autophagosomes caused NS5A levels to decline significantly, while an increase in autophagy flux induced by IL28A overexpression was unaffected by 3-MA, meaning IL28A action occurs after autophagosome formation. Meanwhile, a modest fall of NS5A level was observed in the group of 3-MA without IL28A compared to cells transfected only with NS5A (Fig. 2e), suggesting the fall probably resulted from 3-MA inhibition on autophagosomes. Thus, we infer that IL28A may function in promoting the fusion of autophagosomes with lysosomes.

### The D4 domain of IL28A functions in the formation of autolysosomes

**Fig. 3:**
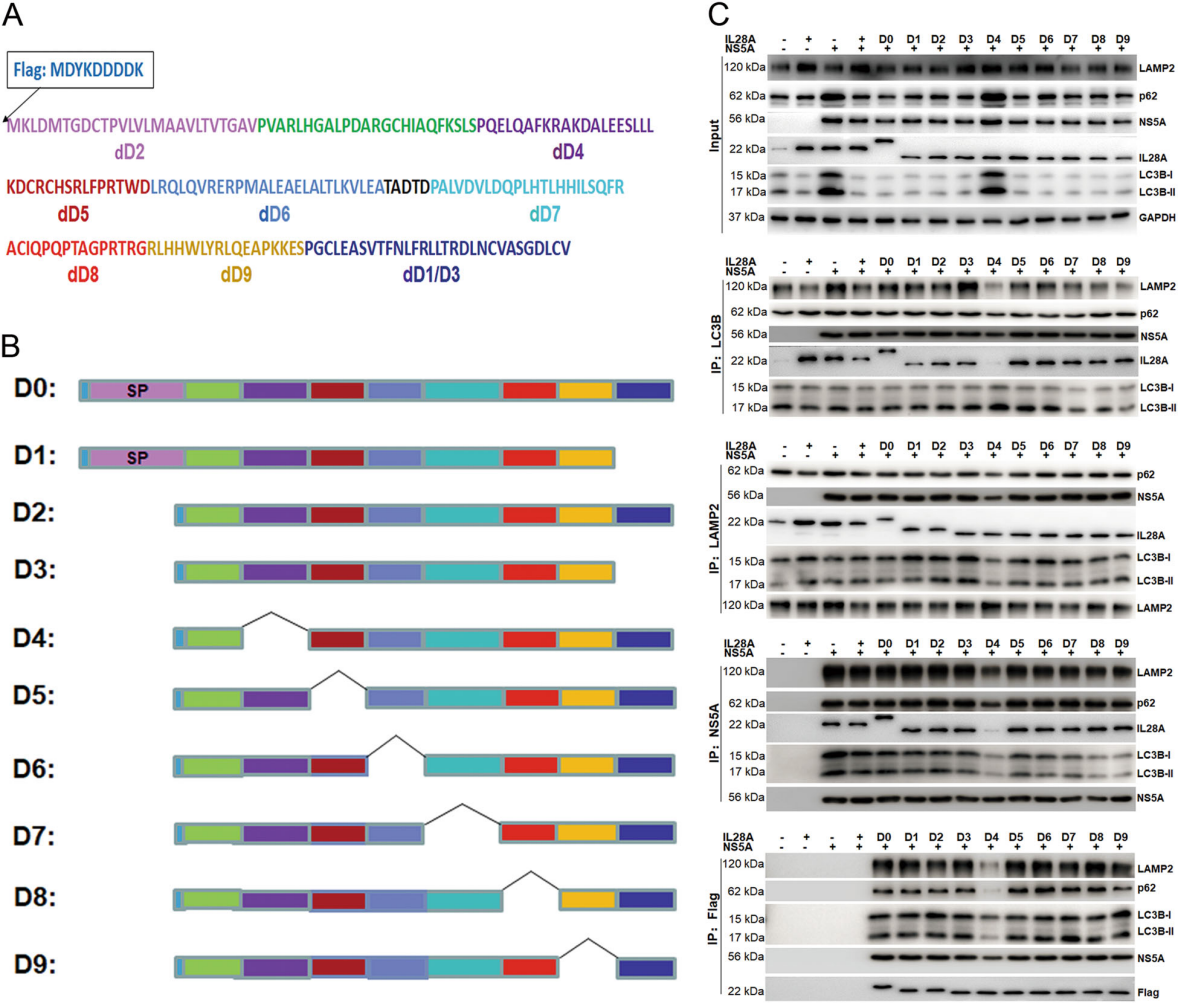
Use of sequential deletions in IL28A to identify the domain responsible for its pro-autophagy activity. **a** IL28A full amino acid sequence showing the names of deleted sequence segments called dD2-dD9 and dD1/dD3. **b** Diagrams of ten Flag-tagged IL28A deletion mutants (D0–D9). **c** Co-immunoprecipitation was used to search a domain required for IL28A function in promoting autolysosome formation in HCV-NS5A expressing HepG2 cells. HepG2 cells were co-transfected with HCV-NS5A and each IL28A mutant (D0–D9, and WT IL28A, respectively) compared with the cells of no-IL28A overexpression. The IP-antibodies are anti-LC3B, anti-LAMP2, anti-NS5A, and anti-Flag (Flag-IL28A). The results showed that only dD4 sequence deletion (D4 group) inhibited the autolysosome formation and increased NS5A protein output compared with D0 group.

We used a sequential deletion method to identify specific domains of IL28A that are indispensable for its action in autophagy and digesting the HCV NS5A protein. We constructed nine sequential deletion constructs of Flag-IL28A, D1–D9 (Fig. 3a, b). These nine deletion mutants of IL28A were transfected separately into NS5A-HepG2 cells. Potential target domains were examined using Co-IP test. In the input assay, protein levels of NS5A, p62, and LC3B were decreased, meaning autophagy flux opened in the cells expressing wildtype (WT) IL28A or flag-labeled IL28A-D0, -D1, -D2, -D3, -D5, -D6, -D7, -D8, and -D9, but not IL28A-D4, compared to the control group of NS5A alone (Fig. 3c). Interestingly, in the co-immunoprecipitation experiments using LAMP2 or LC3B antibodies, when IL28A-D4 was overexpressed, complexes of LAMP2 with both LC3B and p62 were reduced greatly, indicating a decrease in the autophagosome fusion with lysosomes compared to the other IL28A mutant groups (Fig. 3c). Similarly, levels of NS5A and IL28A complexed with LC3B and with LAMP2 were also reduced in D4 overexpression group. In addition, when immunoprecipitated with NS5A antibody, levels of LC3B, LAMP2, p62, and IL28A proteins were substantially lower in the IL28A-D4 group than in the other IL28A mutant groups (Fig. 3c), which may mean that most of NS5A was not bound to IL28A and autophagosomes, and therefore would not be degraded by lysosomes. Importantly, when immunoprecipitated with Flag antibody (representing IL28A), levels of NS5A and the autophagy markers LC3B, p62, and LAMP2 were significantly reduced in the D4 group (Fig. 3c). These results implied that the deleted sequence in IL28A-D4 contributes to the binding site on the proteins involved in autophagolysosome formation, and to its binding on NS5A protein (Fig. 3c). Thus, the deleted sequence in IL28A-D4 is likely to be a critical domain for IL28A in its role of promoting autolysosome formation.

**Fig. 4:**
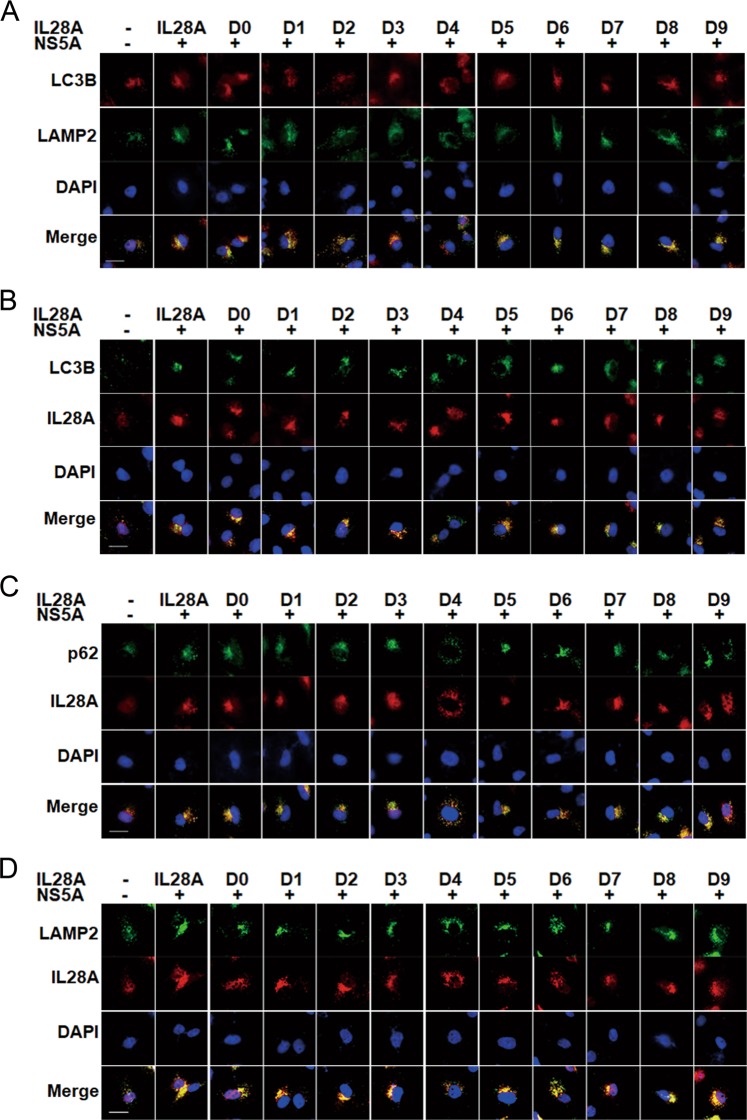
Effects of sequential deletions in the IL28A protein on the subcellular colocalization of autophagosomes with lysosomes and with the IL28A mutants. The nine deletion mutants of IL28A were separately transfected into NS5A-HepG2 cells, and co-localization of the following pairs of proteins were detected by double immunofluorescence staining as follows: **a** LC3B and LAMP2 (autophagosome with lysosome co-localization); **b** autophagosome (LC3B) and IL28A mutants; **c** p62 and IL28A mutants; and **d** LAMP2 (lysosomes) with IL28A mutants. Only the D4 group showed a substantial reduction in particles and Pearson coefficient of co-localization of the protein pairs and altered distribution of proteins. DAPI staining indicates cellular nuclei. The Pearson coefficients can be seen in Fig. S3. Scale bars = 15 μm.

Further, we confirmed the above results using cellular immunofluorescence assay. The results showed that overexpression of IL28A WT, D0, D1, D2, D3, D5, D6, D7, D8, or D9 constructs increased the number of co-localization particles in which LC3B was associated with LAMP2, IL28A was associated with LC3B, with LAMP2, and with p62, in the NS5A-expressed cells. Conversely, the particles containing the co-localized proteins disappeared or were diminished in number in cells expressing IL28A-D4 (Fig. 4a–d). These results indicate the D4 sequence of IL28A being an important functional domain for the promotion of autolysosome formation by IL28A.

### The recognition of NS5A by IL28A is mediated by the NS5A ISDR domain

**Fig. 5:**
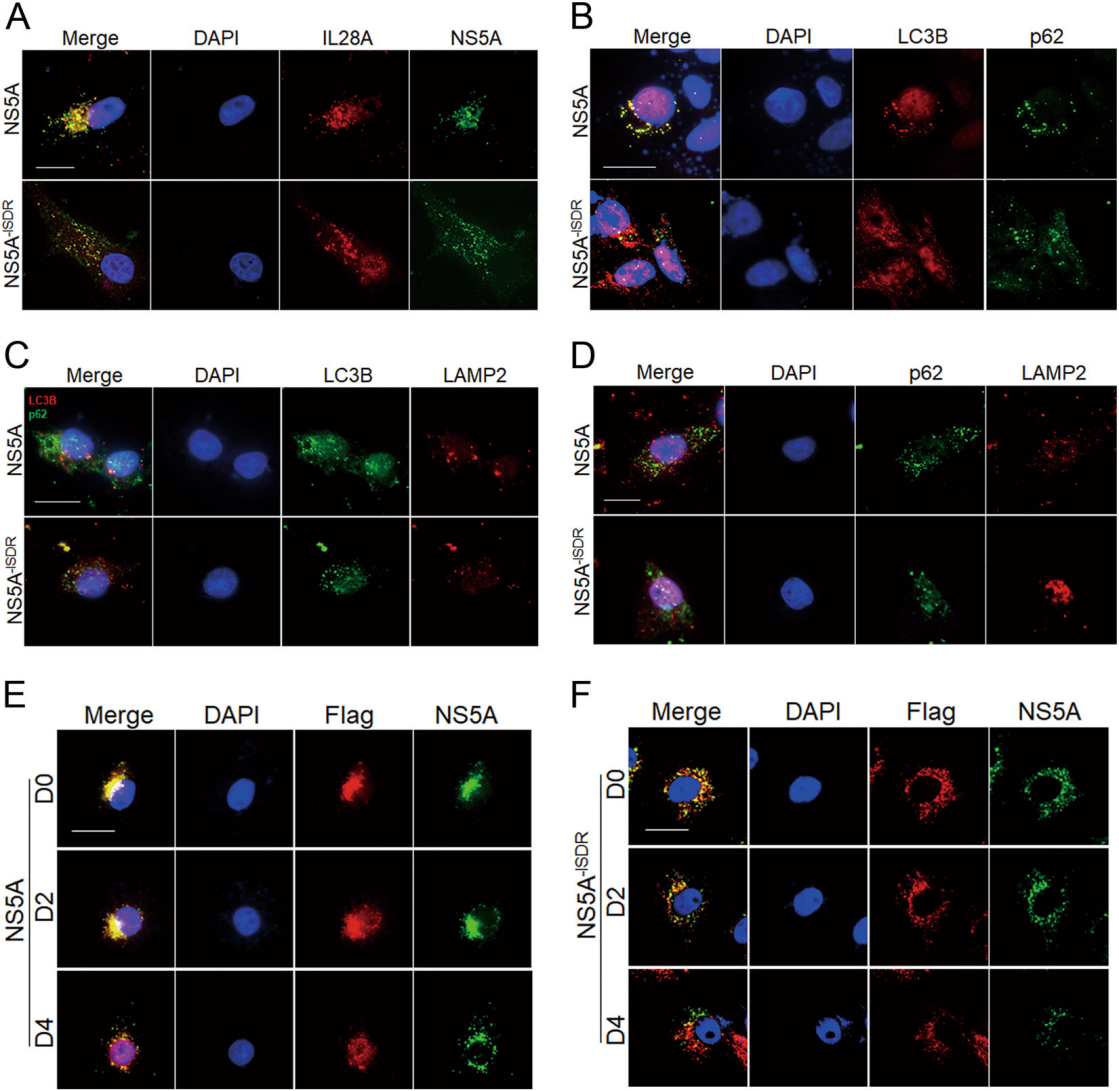
Effects of deleting the ISDR domain of HCV NS5A on autolysosome formation and on the combination of NS5A with IL28A/IL28A mutants. Gene expression plasmids of the NS5A WT and its ISDR deletion mutant were separately transfected into HepG2 cells. Co-localizations of the following pairs of proteins were observed by cell double immunofluorescence imaging. **a** Co-localization of HCV-NS5A or HCV-NS5A^-ISDR^ with IL28A was detected using anti-hIL28A and anti-HCV-NS5A antibodies. **b–d** Co-localizations of autophagy flux related marker proteins were determined using their corresponding antibodies, LC3B with p62 (**b**), LAMP2 with LC3B (**c**) and LAMP2 with p62 (**d**). **e**, **f** IL28A mutation disturbed the interaction between the IL28A and HCV-NS5A/NS5A^-ISDR^. Co-localizations of IL28A mutant D0, D2 or D4 with wildtype NS5A or with NS5A^-ISDR^ were examined via D0, D2, or D4 were co-transfected with the WT NS5A expression construct (**e**) or with the ISDR deletion construct (**f**), using anti-Flag antibody (the IL28A mutants were labeled with the flag) and anti-HCV-NS5A antibody. The scatter diagrams of co-localization particles of IL28A-NS5A, LC3B-p62, LC3B-LAMP2, p62-LAMP2, NS5A-Flag, and NS5A^-ISDR^-Flag, and their Pearson coefficients can be seen in Fig. S4. Scale bars = 15 μm.

Then, we examined whether NS5A ISDR domain also interacts with type III interferon IL28A, and whether the IL28A-D4 domain is involved in the interaction. We tested the interaction between NS5A and IL28A proteins using an NS5A^-ISDR^ deletion mutant and the IL28A-D4 deletion mutant with double immunofluorescence staining. The results showed that colocalized particles of WT NS5A with IL28A were found at the perinuclear region, but NS5A^-ISDR^–IL28A interaction was weakened in both the particle number and Pearson co-localization coefficient (Fig. S4A), and the position of the particles shifted from the perinuclear region to the cytoplasm widely (Fig. 5a), indicating that the ISDR domain has an essential role in recognizing and binding to IL28A, as with type 1 interferon. The number of co-localized particles of LC3B with p62 were significantly reduced in the NS5A^-ISDR^ groups compared with the WT NS5A groups (Fig. 5b) and the associations of LAMP2 with LC3B and with p62 proteins still were not occurred as the same as in the WT NS5A group (Fig. 5c, d).

Next, we evaluated the effects of the ISDR domain and the dD4 sequence on the interaction using flag-labeled IL28A mutants and NS5A^-ISDR^. Particles of the flag-IL28A mutants colocalized with NS5A could be seen clustered near one side of the nucleus in the IL28A-D0, -D2, and -D4 groups, but were reduced considerably in particle number and Pearson co-localization coefficient in IL28A-D4 group (Figs. 5e and S4E). Under conditions of NS5A^-ISDR^ overexpression, the numbers of colocalized particles of NS5A^-ISDR^ with the IL28A mutants were significantly less than those of the NS5A groups and the particles scattered around the nucleus in the IL28A-D0 and -D2 groups. In the IL28A-D4 group, hardly any colocalized particles were detected (Figs. 5f and S4F). These findings suggested that the ISDR domain of NS5A is crucial for direct association with the IL28A protein, and its absence affected the intracellular particle distribution. The deletion of the dD4 sequence in IL28A further prohibits the association between IL28A and HCV NS5A proteins, leading to their complete dissociation from one another (Fig. 5f).

### The IL28A homotetramer functions in the promotion of autophagolysosome formation

**Fig. 6:**
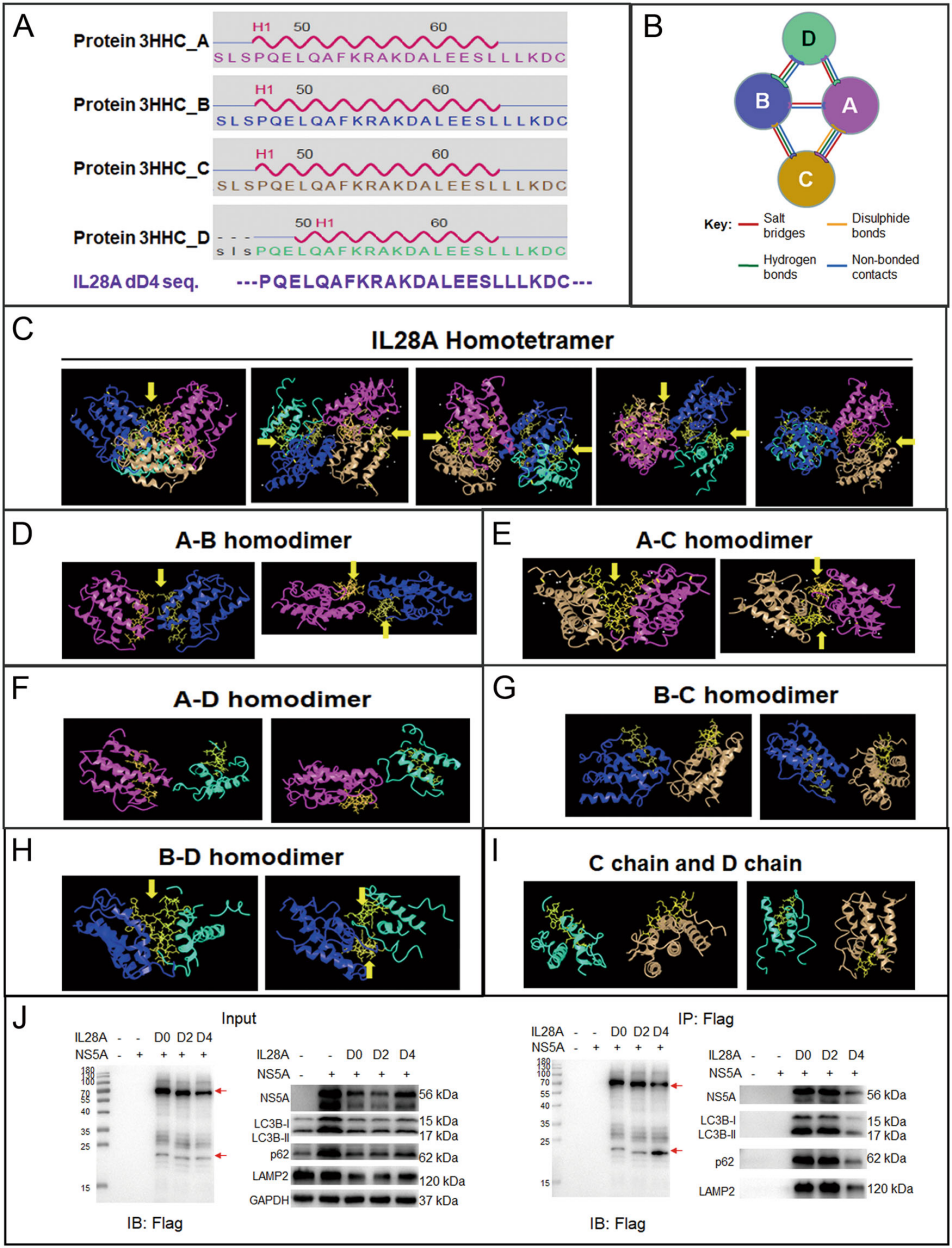
Prediction and confirmation of the structure of the IL28A homotetramer. **a** The sequence of the first α-helices in chains A, B, C, and D of IL28A (top four panels), from the website (wamhttps://www.ncbi.nlm.nih.gov/Structure/icn3d/full.html?&mmdbid=75484&bu=0&showanno=1), which show the sequence of the first α-helix (H1) exactly corresponding to the deleted sequence dD4 (bottom) in the D4 mutant. **b** A diagram showing the interfaces among the four chains of IL28A homotetramer which was drawn based on the figure on the website (http://www.ebi.ac.uk/thornton-srv/databases/cgi-bin/pdbsum/GetPage.pl?pdbcode=3hhc&template=interfaces.html&c=999.). The four balls represent the four chains of IL28A (**a–d**) in different colors that match the corresponding chains in (**c**–**i**). **c** Conformations of the IL28A homotetramer viewed from different visual angles. **d**, **e**, **h** The first α-helix contributes to the structure of the binding sites at the interfaces between three pairs of chains (A:B, A:C, and B:D; see the yellow arrows); **f**, **g** bonding between the two pairs of chains (A:D, B:C) may not be dependent on the first α-helix; and **i** no bonding between the C chain and D chain occurs according to the prediction online (The prediction and the conformation figures are derived from the website https://www.ncbi.nlm.nih.gov/Structure/icn3d/full.html?&mmdbid=75484&bu=0&showanno=1). The yellow chemical structural formulas show the D4 deleted sequence-the first α-helix. Yellow arrows indicate where the first α-helixes participate in interactions. **j** The IL28A homotetramer spatial structure correlating with its functions is confirmed by Co-IP experiment. Input result showed that deletion of the dD4 sequence (D4) reduced the fraction of homotetramer, while the monomer fraction and NS5A increased and the autophagy flux became blocked (p62 increased compared to the D0 group). When immunoprecipitated with anti-flag antibody, the homotetramer fraction of IL28A was again reduced in the D4 group, with a corresponding elevation of IL28A monomer, while the levels of NS5A, LC3B, p62, and LAMP2 decreased. Red arrows point to IL28A tetramer and monomer.

To investigate the molecular mechanism of IL28A anti-HCV effects and pro-autophagy, we attempted to define the structure of IL28A for the IL28A protein functional motif and stereostructure using online software at the ncbi and ebi sites (https://www.ncbi.nlm.nih.gov/Structure/icn3d/full.html?&mmdbid=75484&bu=0&showanno=1; http://www.ebi.ac.uk/thornton-srv/databases/cgi-bin/pdbsum/GetPage.pl?pdbcode=3hhc&template=interfaces.html&c=999). We based the structure prediction on IL28B with 196 amino acid-long, because there is a more than 96% identity between the IL28A and IL28B proteins. The prediction showed that the IL28A conformation might be a homotetramer of the A, B, C, and D chains. Their secondary structures include mainly helixes and coils. There are six helixes and six coils dispersed in the 159 amino acid residues of the A, B, and C chains, and three helixes and four coils in the 159 residues of the D chain. There are three intrachain disulfide bonds in A, B, and C chains, and one intrachain disulfide bond in D chain. There is a single interchain disulfide bond between the A and C chains, (https://www.ncbi.nlm.nih.gov/Structure/icn3d/full.html?&mmdbid=75484&bu=0&showanno=1). Among these conserved domains, the first α-helix corresponds exactly to the deleted sequence dD4 in IL28A-D4 mutant (Fig. 6a). Based on the IL28B homotetramer model, both interface areas between the A and C chains and between the B and D chains are significantly larger than the other three interface areas between pairs of A–B, A–D, or B–C chains (Table 1). There are interchain cross-linkages among the four IL28A molecules, three pairs of chains (A–D, B–C, and B–D) interacting through salt bridges, hydrogen bonds, and non-bonded contacts; the A–B chains bonded by salt bridges and hydrogen bonds; the A–C chains held together by four kinds of bonds: an interchain disulfide bond and the above three kinds of bonds (Fig. 6b; https://www.ncbi.nlm.nih.gov/Structure/mmdb/mmdbsrv.cgi?dps=2&uid=3HHC;^41^). According to the above model, the first α-helix participates in the interfaces between A–B, A–C, and B–D chains (Fig. 6c–e, h), while the linkages between A and D chains and between B and C chains may not be dependent on the first α-helix (Fig. 6c, f, g). No linkage between the C and D chains was predicted (Fig. 6i). Because our previous results showed that the D4 mutant without the first α-helix lost IL28A pro-autophagy activity, we examined whether the deletion of the first α-helix could disrupt the IL28A oligomer structure and result in loss of IL28A function by using Co-IP. Western blot results (input panel) showed that the IL28A protein exhibited mainly two bands of about 70 kDa and 20 kDa, suggesting the existence of IL28A tetramers. Deletion of dD4 sequence in the IL28A-D4 mutant caused a reduced amount of the IL28A homotetramer while NS5A increased, and autophagy flux became blocked (manifested by increased levels of LC3B and p62 proteins compared with D0 and D2). The only difference between D0 and D2 was the presence or absence of the signal peptide; both had the same activity (Fig. 6j, left panel), meaning that the signal peptide did not affect the IL28A function. In co-immunoprecipitation studies using anti-flag antibody, in the “D4-deleted” group, the IL28A homotetramer was also reduced, but the IL28A monomer increased, indicating that the first α-helix is positively associated with IL28A oligomer formation; at the same time, levels of NS5A, LC3B, p62, and LAMP2 decreased. These findings suggested that the interaction between these proteins and the D4-IL28A protein was significantly reduced due to dD4 sequence deletion. The D2 group gave the same results as the D0 group (Fig. 6j, right panel). These results suggest that the tetrameric structure of IL28A may be necessary for its role in promoting autophagolysosome formation and NS5A degradation. They also imply that the first α-helix of IL28A is a critical domain for the interactions between A and B, A and C, and B and D chains associated with the formation and stability of IL28A homotetramers.

**Table 1:** Interface statistics of IL28B homotetramers.

Chains	No. of interface residues	Interface area (Å^2^)
A:B	5:5	277:283
A:C	19:21	1092:1058
A:D	6:5	291:256
B:C	6:8	338:291
B:D	19:18	1124:1116

The Table 1 was downloaded from the website http://www.ebi.ac.uk/thornton-srv/databases/cgi-bin/pdbsum/GetPage.pl?pdbcode=3hhc&template=interfaces.html&c=999.

### IL28A has the same effect on the other HCV proteins

**Fig. 7:**
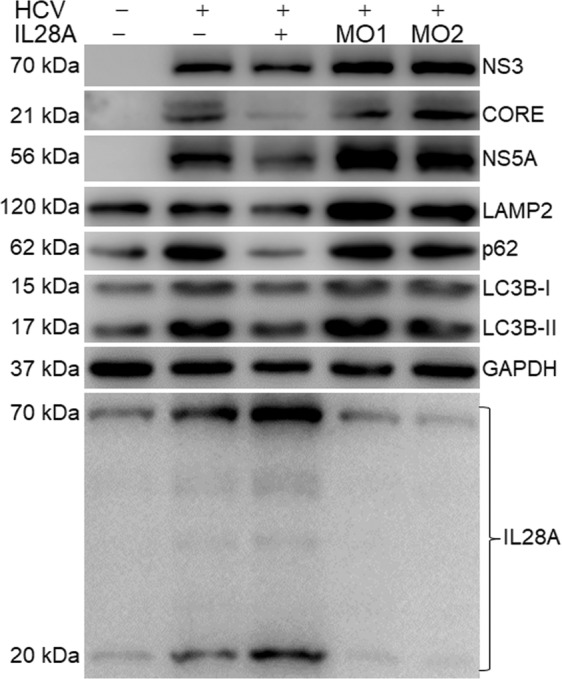
IL28A inhibition on the other HCV proteins in HCV virion infected Huh7.5 cells. Huh7.5 cells were first transfected with designed concentrations of *IL28A* expression construct, IL28A-MO1 and IL28A-MO2, and cultured for 12 h, and then were infected with HCV virion (J6/JFH/JC, 45 IU/cell) for 72 h. The cells were collected for detection with western blotting. HCV NS3, CORE, and NS5A as the representatives of HCV proteins were determined and their levels were oppositely correlative with the IL28A levels. IL28A image shows IL28A homotetramer and monomer.

To verify IL28A protein effect on the other HCV proteins, we examined the HCV NS3, CORE, and NS5A proteins (as the representative of full HCV proteins) and autophagy flux-related proteins, LC3B, p62, and LAMP2 using the full HCV virion (J6/JFH/JC) infected Huh7.5 cells. The results show that IL28A overexpression caused significant decrease of HCV NS3, CORE, and NS5A proteins along with an unimpeded autophagy flux. Meanwhile, IL28A homotetramer appeared dominant compared with its monomer. IL28A downregulation by morpholino oligoes (MOs) blocked autophagy flux and elevated obviously or moderately the levels of the HCV proteins compared with the HCV virion group (Fig. 7). These results suggest that IL28A has the same inhibition on the other HCV proteins as on NS5A expression alone.

## Discussion

Previous studies reported that HCV NS3, NS4A, NS4B, NS5A, and NS5B proteins formed a complex to mediate the replication of the HCV genomic RNA^37^. The HCV NS5A protein is an important component of the HCV RNA replication complex and directs the replication complex docking to autophagosome membranes^37^. The N-terminal 30 amino acids of NS5A have been predicted to form a highly conserved amphipathic α-helix that is both necessary and sufficient for mediating the association of NS5A with the ER membrane/autophagopore membrane, which facilitates the adherence and replication of the HCV replication complex there^42^. These studies suggested that autophagy can benefit HCV replication. Our studies indicated that HCV NS5A indeed enhanced autophagosome formation by activating the proteins ATG3, ATG5, ATG7, and ATG10 of the ubiquitin-like system. These ubiquitin-like proteins can facilitate the transformation of LC3B-I to LC3B-II and the increase of the autophagosomes. The immunofluorescence assays reported here that HCV NS5A protein became associated with LC3B and p62, but not with LAMP2, meaning that NS5A promoted autophagosome formation to recruit the intracellular membranes for the HCV replication complex resided but inhibited fusion of autophagosomes to lysosome to avoid lysosomal degradation of the HCV products. Thus, HCV NS5A can exploit host autophagy machinery to help HCV replication.

However, many studies have described the synergistic action of interferons (mainly, type I interferons) with autophagy and other host factors in fighting viruses^15–22^. But few reports have been published on the synergistic effects of type III interferons with autophagy against viruses. In our previous studies, we found that IL28A inhibited the replication of the HCV sub-genome by promoting autolysosome formation in vitro^39,40^. In this study, we found that IL28A can degrade NS5A through the promotion of autolysosome formation and lysosomal degradation, which is supported by Yoo et al.^43^. Furthermore, we researched the direct interaction between NS5A and IL28A. The ISDR of NS5A was reported previously to function as the domain for recognizing and binding type I interferon, by which NS5A inhibits the interferon signaling pathway to facilitate HCV replication^44^. Here, we demonstrate that IL28A can also interact with HCV NS5A through the ISDR domain. We speculate that high expression of IL28A may degrade HCV NS5A; and high level of HCV NS5A may suppress the action of IL28A. The details in the combat process remain unclear, but the result of combat between IL28A and HCV NS5A will significantly affect the HCV replication. Thus, activation of IL28A expression may be a new strategy for the HCV antiviral therapy.

**Fig. 8:**
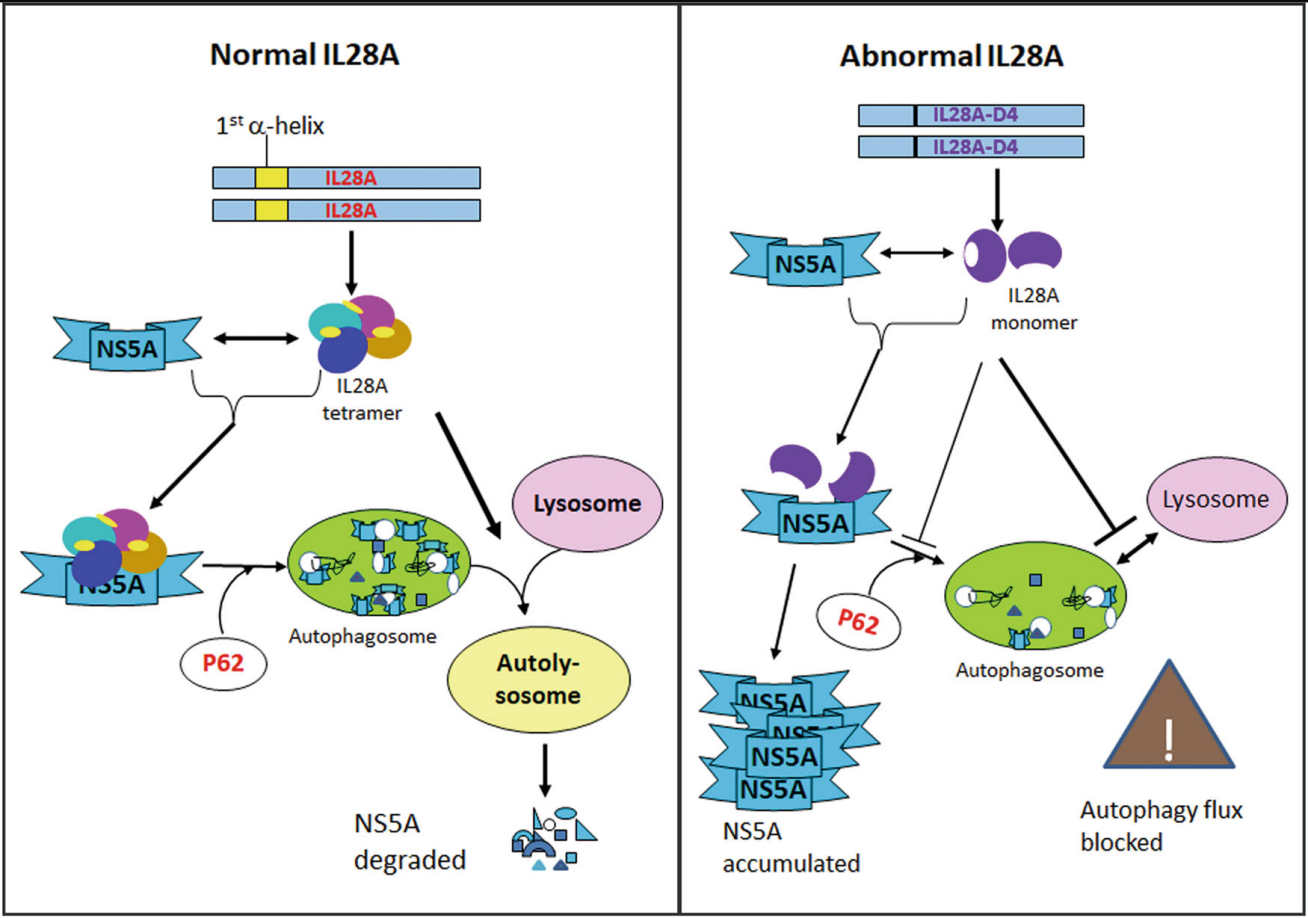
A working model for the function of the IL28A homotetramer. Normally IL28A protein plays its roles in interacting with HCV proteins and promoting fusion of autophagosomes to lysosomes by its homotetramer conformation. Deletion of the firs a-helix of IL28A protein causes the homotetramer disintegrated and IL28A failed to recognizing NS5A and to promoting formation of autolysosomes.

Until now, research on the molecular mechanism of interferons’ action including IL28A, has focused largely on their downstream signaling pathways, receptors and the activation of immunity-related genes. There is no study on the correlation of structure with function in IL28A. Respecting that protein conformation is closely related to its function, we modeled the three-dimensional conformation of IL28A on the previously described structure of IL28B (https://www.ncbi.nlm.nih.gov/Structure/icn3d/full.html?&mmdbid=75484&bu=0&showanno=1), the sequence of IL28B is 96% similar to that of IL28A. The structural modeling showed that IL28A is likely to be in a homotetrameric form and that the first α-helix in each molecule is situated at the interfaces among three pairs of the four chains, such as A–B, A–C, and B–D chains, to contribute to conjugation of IL28A homotetramer. The results of experiments with IL28A deletion mutants demonstrated that HCV NS5A degradation was positively correlated with levels of the IL28A tetramer, rather than its monomer (Fig. 6j). The first α-helical sequence of IL28A was found to be a key domain for the conformation of the homotetramer and for the interaction of IL28A with the autophagic proteins LC3B, p62, and particularly LAMP2. Furthermore, we found that NS5A was degraded by autolysosomes, mediated by the IL28A tetramer. In contrast, when the first α-helix of IL28A was missing, the tetramer disappeared or reduced, and this was followed by the elevation of NS5A levels. We propose that the IL28A homotetramer is an essential structure needed for the direct or indirect association with NS5A, autophagosomes, and lysosomes together; and that the first α-helix of IL28A is required for this protein homotetrameric conformation (Fig. 6). This result has been confirmed via the experiment of the full HCV virion infection (Fig. 7). Our work here presents the first experimentally supported correlation of the IL28A tetramer conformation with its functions (Fig. 8).

In summary, though virus can acitivate an incomplete autophagy pathway to support its survival, upregulation of host defense system such as IL28A can eliminate the virus at the final step of autophagy pathway via promoting autolysosome formation and lysosomal degradation. For the first time, we have demonstrated that IL28A is directly bound to NS5A and that IL28A homotetramer is a key structure for IL28A function in degrading HCV proteins by promoting autolysosome formation. The first helix of IL28A has a pivotal function in maintaining the IL28A homotetramer conformation.

## Materials and methods

### Reagents and antibodies

Lipofectamine 2000 Reagent was purchased from Invitrogen (Cat# 1667501, Carlsbad, CA, America). Protein extracting reagent RIPA lysis buffer (Cat# C1053), non-denaturing lysis buffer (Cat# C1050) and protease inhibitor (cocktail, 50×, Cat# P1265-1) were purchased from Applygen Technologies, Inc (Beijing, China). For Western blotting, anti-NS5A antibody (Cat# ab13833), anti-NS3 antibody (Cat# ab13830), anti-core antibody (Cat# ab2740), anti-IL28A antibody (Cat# ab38570), and anti-DDDDK tag antibody (Cat# ab1162) were purchased from Abcam (Cambs, UK); anti-p62 (Cat# PM045), anti-LC3B (Cat# M186-3) were purchased from MBL (Japan). Antibodies against ATG3 (Cat# AP1807b), ATG5 (Cat# AP1812a) and ATG10 (Cat# A9356) were purchased from Sigma-Aldrich (America). Anti-ATG7 (Cat# sc-33211) and anti-LAMP2 (Cat# sc-18822) were purchased from Santa Cruz Biotechnology (America). Anti-GAPDH (Cat# TA-08) and horseradish peroxidase-(HRP)-conjugated goat anti-mouse IgGs (Cat# ZB2305) and goat anti-rabbit IgGs (Cat# ZB2301) were purchased from ZSGB-BIO Co. (Beijing, China). For immunoprecipitation, anti-LC3B (Cat# PM036) and anti-p62 (Cat# PM045) were purchased from MBL (Japan), anti-NS5A (Cat# ab20342) and anti-DDDDK tag antibody (Cat# ab1162) from Abcam (America), and anti-LAMP2 (Cat# sc-18822) from Santa Cruz Biotechnology (America). Rabbit IgG (Cat# 58802 S) and mouse IgG (Cat# 93702 S) were purchased from Cell Signaling Technology (CST, Danvers, MA, America); protein A/G plus-agarose (Cat# sc-2003) was obtained from Santa Cruz Biotechnology (America). For cellular immunofluorescence and co-localization experiments, anti-NS5A antibody (Cat# ab12833), Human IL-28A antibody (Cat# ab191426), anti-LAMP2 (Cat# ab13524), anti-DDDDK tag antibody (Cat# ab1162) and anti-p62 antibody (Cat# ab56416) were purchased from Abcam (Cambs, UK). Anti-LC3B (Cat# PM036) and anti-p62 (Cat# PM045) were purchased from MBL (Japan). The TRITC (tetramethyl rhodamine isothiocynate) labeled goat anti-rabbit IgG secondary antibodies (Cat# ZF-0316), FITC (fluorescein isothiocyanate)-labeled goat anti-mouse IgG secondary antibodies (Cat# ZF-0312) and mounting medium with DAPI (4’,6-diamidino-2-phenylindole, Cat# ZLI-9557) were purchased from ZSGB-BIO (Beijing, China).

### Cell line and mock-transfected cells

HepG2 cells were purchased from the National Infrastructure of Cell Line Resource. Cells were cultured in MEM (Gibco, America) supplemented with 10% fetal bovine serum at 37 °C in a 5% CO_2_ incubator. A mock group was obtained by pIRES2-EGFP transfection into HepG2 cell.

### Plasmids

The complete coding sequence of HCV NS5A was subcloned from HCV J4L6S (1b) (stored in our lab) using PCR and inserted into pIRES2-EGFP vector to obtain the HCV NS5A overexpression construct pIRES2-NS5A. The NS5A^-ISDR^ deletion mutant was constructed by overlap extension PCR in pIRES2-EGFP vector as pIRES2-NS5A^-ISDR^. The IL28A deletion mutants were designed based on the prediction of human IL28A protein secondary structure online (http://www.ebi.ac.uk/thornton-srv/databases/cgi-bin/pdbsum/GetPage.pl?pdbcode=3hhc&template=protein.html&r=wiring&l=1&chain=A) and shown in Fig. 3a, b. The internal deletion mutants IL28A-D4, IL28A-D5, IL28A-D6, IL28A-D7, IL28A-D8 and IL28A-D9 in pIRES2-EGFP were synthesized by Sangon Biotech Co. (Shanghai, China). The 5′ and 3′ flanking region mutants IL28A-D0, IL28A-D1, IL28A-D2 and IL28A-D3 in pIRES2-EGFP vector were constructed using PCR with specific primers at the designed sequence sites. All the expression constructs of IL28A-derived gene mutants and the wild type were Flag-tagged at their N-terminals and identified by sequencing.

### Overexpression of IL28A-derived gene mutant constructs in cells expressing NS5A

Transient transfection was performed using Lipofectamine 2000 reagent according to the manufacturer’s instructions. First, HepG2 cells were transfected with pIRES2-NS5A and cultivated for 12 h, then re-transfected with each IL28A-derived mutant plasmid, respectively, for 48 h culture and collected for subsequent experiments.

### Downregulation of IL28A by morpholinos in cells overexpressing NS5A

The IL-28A morpholino oligomer sequences are 5′-TTCATTCCT GATCTCTGGTCTTTGT-3′ (MO1), and 5′-AAACACTCTGAGGCTGTCACCCAGG-3′ (MO2) bought from Gene Tools, LLC. (MO1 covers the start codon ATG, and MO2 is located at the ribosomal binding site and is 29 bases apart from the start codon ATG) which can downregulate the human IL28A protein in target cells. IL28A knockdown was carried out by the morpholino transfection at concentrations of 100 pM for each well in cells expressing NS5A. Then, the cells were cultured for 48 h and collected for the succeeding detection using both Co-IP and cell immunofluorescence methods.

### Influence of autophagy inhibitors on IL28A function in autophagy flux in cells overexpressing NS5A and IL28A

HepG2 cells were transfected with HCV NS5A and cultured for 12 h, then re-transfected with IL28A for 24 h. The cells were then exposed to 3-MA (1 mM, Sigma-Aldrich) or CQ (50 μM, Sigma-Aldrich) for another 24 h. The cells were collected and broken, and the cell supernatants were examined for changes in the levels of HCV NS5A, LAMP2, p62, and LC3BII/I using western blots.

### Western blotting and co-immunoprecipitation

Proteins were extracted with RIPA lysis buffer from cells treated as described above and separated on the SDS-PAGE. The proteins were transferred onto a nitrocellulose membrane by blotting. The membranes were incubated separately with anti-NS5A, anti-p62, anti-LC3B, anti-LAMP2, anti-IL28A, anti-Flag and anti-GAPDH at a dilution range of 1:1000 to 1:2000 in TBST (mixture of tris-buffered saline and Tween 20) at 4 °C overnight. The membranes were then washed and incubated with HRP-conjugated goat anti-mouse or goat anti-rabbit IgGs (1:2000 dilutions) for 1 h at room temperature. Proteins were detected using the chemiluminescent HRP substrate (Millipore) with Tanon-5200 FC Imaging System (Tanon Science & Technology Co. Ltd., China). The optical intensities were quantified by Gel-Pro analyzer.

For immunoprecipitation, cells were harvested and lysed with nondenaturing lysis buffer and protease inhibitor cocktails. After pre-binding with protein A/G agarose-plus beads for 1 h at 4 °C, whole-cell lysates were used for immunoprecipitation with the indicated antibodies. Generally, the 1–2 μg designated antibody was added to cell lysates and incubated at 4 °C overnight. Then the mixtures were added to protein A/G plus agarose beads for 2 h, the immunoprecipitates extensively washed 5 times with PBS and eluted with SDS loading buffer by boiling for 5 min (minutes). The co-precipitates were examined by running SDS-PAGE and Western blotting with suitable antibodies.

### Cell Immunofluorescence experiments

The experimental procedure, in general, followed our previous procedure^39^. Cells were fixed with 1% paraformaldehyde for 15 min at room temperature. After washing three times with PBS, cells were permeabilized with 3% Triton X-100 for 10 min, then probed with anti-p62, anti-LC3B, anti-LAMP2, anti-Flag, anti-NS5A or anti-IL28A antibodies at 4 °C overnight. After 3 washes with PBS, the cells were incubated with secondary antibodies labeled with TRITC or FITC (1:100 dilutions) for 1 h. Next, the cells were counterstained with DAPI dye in mounting medium and observed under a DeltaVision Imaging System (GE Healthcare).

For the study of comparison between HCV-NS5A and HCV-NS5A^-ISDR^ (Fig. 5a) human IL28A antibody was used to detect co-localization of IL28A with HCV-NS5A proteins. The anti-flag antibody was used to avoid the IL28A mutation disturbance on the interaction between the IL28A mutants (the IL28A proteins labeled with flag) and HCV-NS5A^-ISDR^ (Fig. 5e, f).

### HCV virion infection

HCV virion infection was performed as previously described^39^. Huh7.5 cells were transfected with designed concentrations of IL28A, IL28A-MO1, and IL28A-MO2 using Lipofectamine 2000. After 12 h, the culture supernatants were replaced with fresh complete cultural media, and the transfected cells were then infected with HCV virion (J6/JFH/JC, 45 IU/cell) for 72 h. Total proteins and RNAs were extracted and detected with WB and qRT-PCR, repectively^45^.

### Data and statistical analysis

Statistical analysis was performed using GraphPad Prism 5 software. Data shown are mean ± SD; the means and standard deviations in histograms and scatter diagrams are derived from three independent experiments. The one-way analysis of variance (ANOVA) test was used for all data sets and *P*-values <0.05 were considered as significant.

## Supplementary information

Supplementary Figure Legends

Figure S1

Figure S2

Figure S3

Figure S4
