# Supplementary material for: IL28A protein homotetramer structure is required for autolysosomal degradation of HCV-NS5A in vitro
Source: Cell Death Dis. 2020 Mar 23;11(3):200. doi: 10.1038/s41419-020-2400-9 (PMC7090004; doi:10.1038/s41419-020-2400-9)
Supplement: Supplementary file 1 — Supplementary Figure Legends [file 41419_2020_2400_MOESM1_ESM.docx]

**Figure S1. Incomplete autophagy induced by the HCV NS5A protein in HepG2 cells.** (A) Histograms of relative levels of ATG3, ATG5, ATG7, ATG10, LC3B-II/I and p62 proteins by Western blot. *P<0.05, **P<0.01, ***P<0.001. All of the data are mean ± SD (n = 3). (C-H) Cell Immunofluorescence experiments for detecting the effects of HCV NS5A on the subcellular co-localization among autophagosomes of LC3B, substrate receptor (p62), lysosomes (LAMP2), and HCV NS5A. Scatter diagrams show the Pearson coefficients of the two associated protein co-localization and the number of co-localized particles per cell. *P<0.05, **P<0.01, ***P<0.001. The results indicated that NS5A promoted the interaction of LC3B with p62 (C) but not of LAMP2 with LC3B (D) and not with p62 (E). Also, an association between NS5A with p62 (F) and with LC3B (G), but not with LAMP2 (H) was observed. Scale bars = 15μm.

**Figure S2. IL28A promotes autolysosome co-localization with HCV NS5A protein.** Cell Immunofluorescence experiments show that IL28A overexpression promoted the subcellular co-localization of LC3B-LAMP2 (B), LC3B-p62 (C) and NS5A-LAMP2 (D); conversely, IL28A downregulation by MOil28as inhibited the co-localization of these proteins. Scale bars = 15μm. Scatter diagrams show the Pearson coefficients of the two associated protein co-localization and the number of co-localized particles per cell. *P<0.05, **P<0.01, ***P<0.001.

**Figure S3. Effects of sequential deletions in the IL28A protein on the subcellular colocalization of autophagosomes with lysosomes and the IL28A mutants.** Cell Immunofluorescence experiments (Figure 4) show that D4 group showed a substantial reduction in particles of LC3B and LAMP2 (A), LC3B and IL28A (B), and p62 and IL28A (C), and LAMP2 and IL28A (D). *P<0.05, **P<0.01, ***P<0.001.

**Figure S4. Effects of deleting the ISDR domain of NS5A on autolysosome formation and the combination of NS5A with IL28A.** Comparison between NS5A and NS5A^-ISDR^ in co-localization Pearson coefficients and the number of co-localized particles per cell were resulted from the cell immunofluorescence imaging (Figure 5): NS5A and IL28A (A), LC3B and p62 (B), and LAMP2 and LC3B (C), LAMP2 and p62 (D), NS5A and IL28A (E), and NS5A^-ISDR^ and IL28A (F). *P<0.05, **P<0.01, ***P<0.001.
