# Supplementary figures and images for: IL28A protein homotetramer structure is required for autolysosomal degradation of HCV-NS5A in vitro
Source: Cell Death Dis. 2020 Mar 23;11(3):200. doi: 10.1038/s41419-020-2400-9 (PMC7090004; doi:10.1038/s41419-020-2400-9)

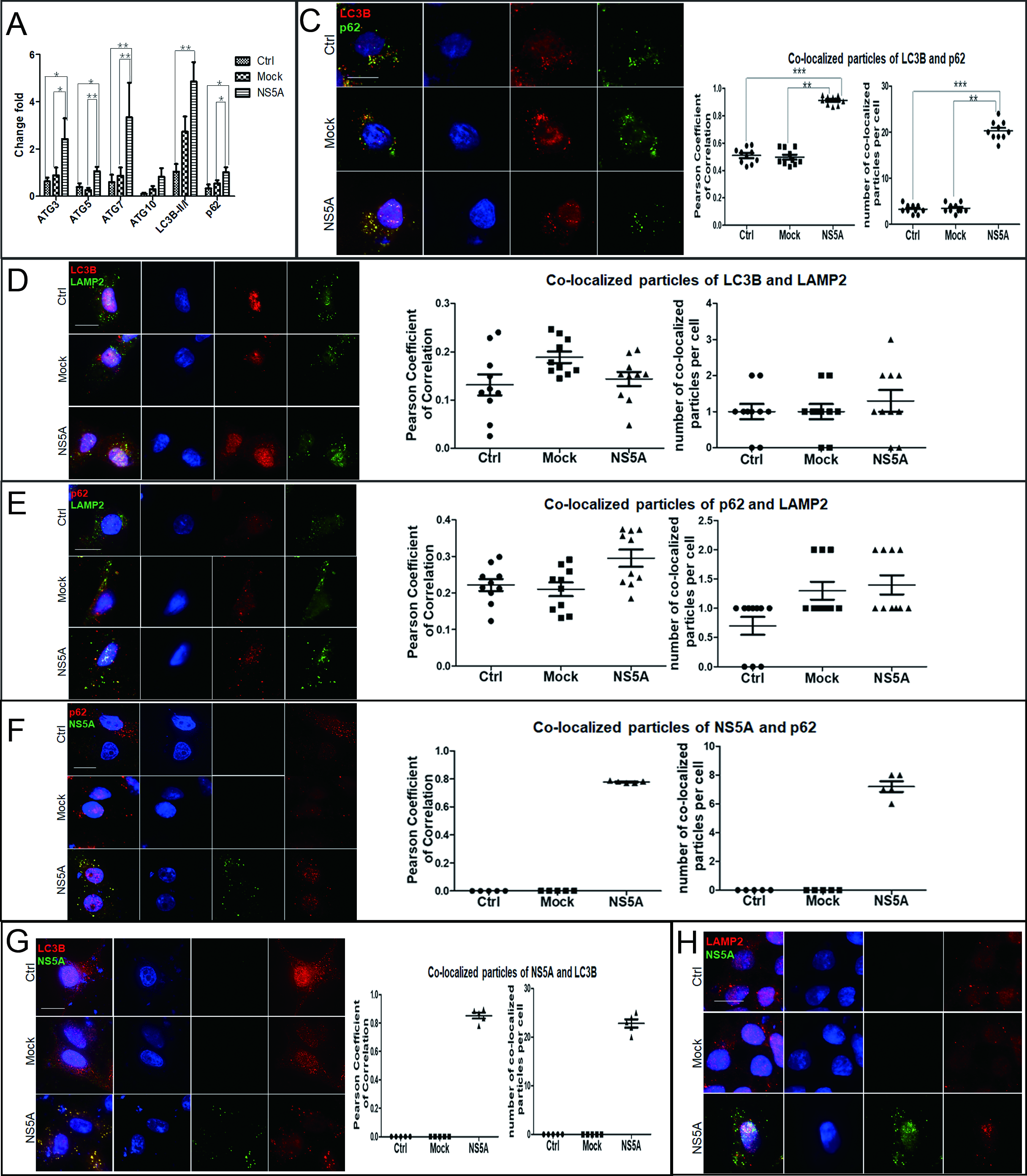

Supplement: Supplementary file 2 — Figure S1 [file 41419_2020_2400_MOESM2_ESM.tif]

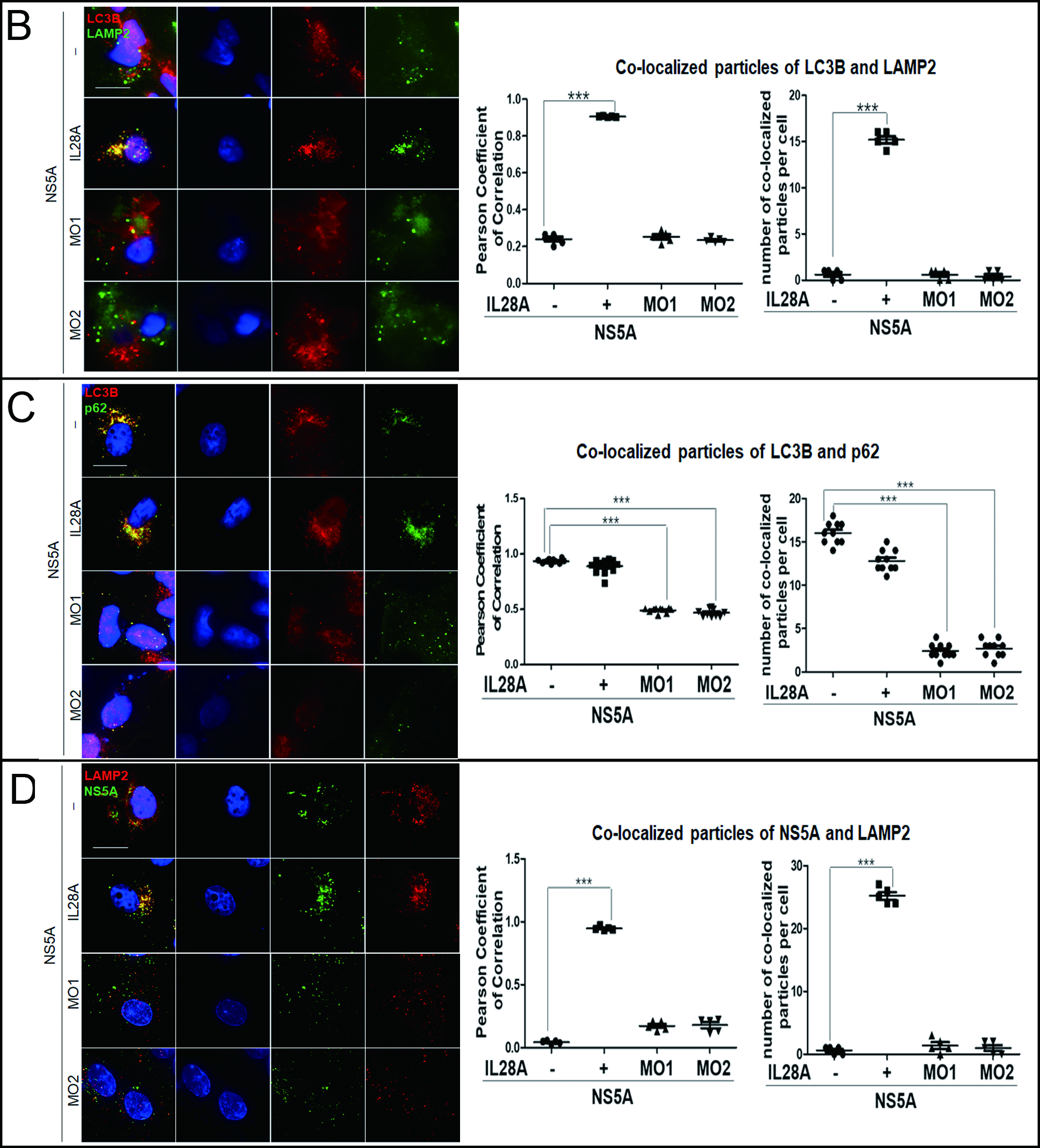

Supplement: Supplementary file 3 — Figure S2 [file 41419_2020_2400_MOESM3_ESM.tif]

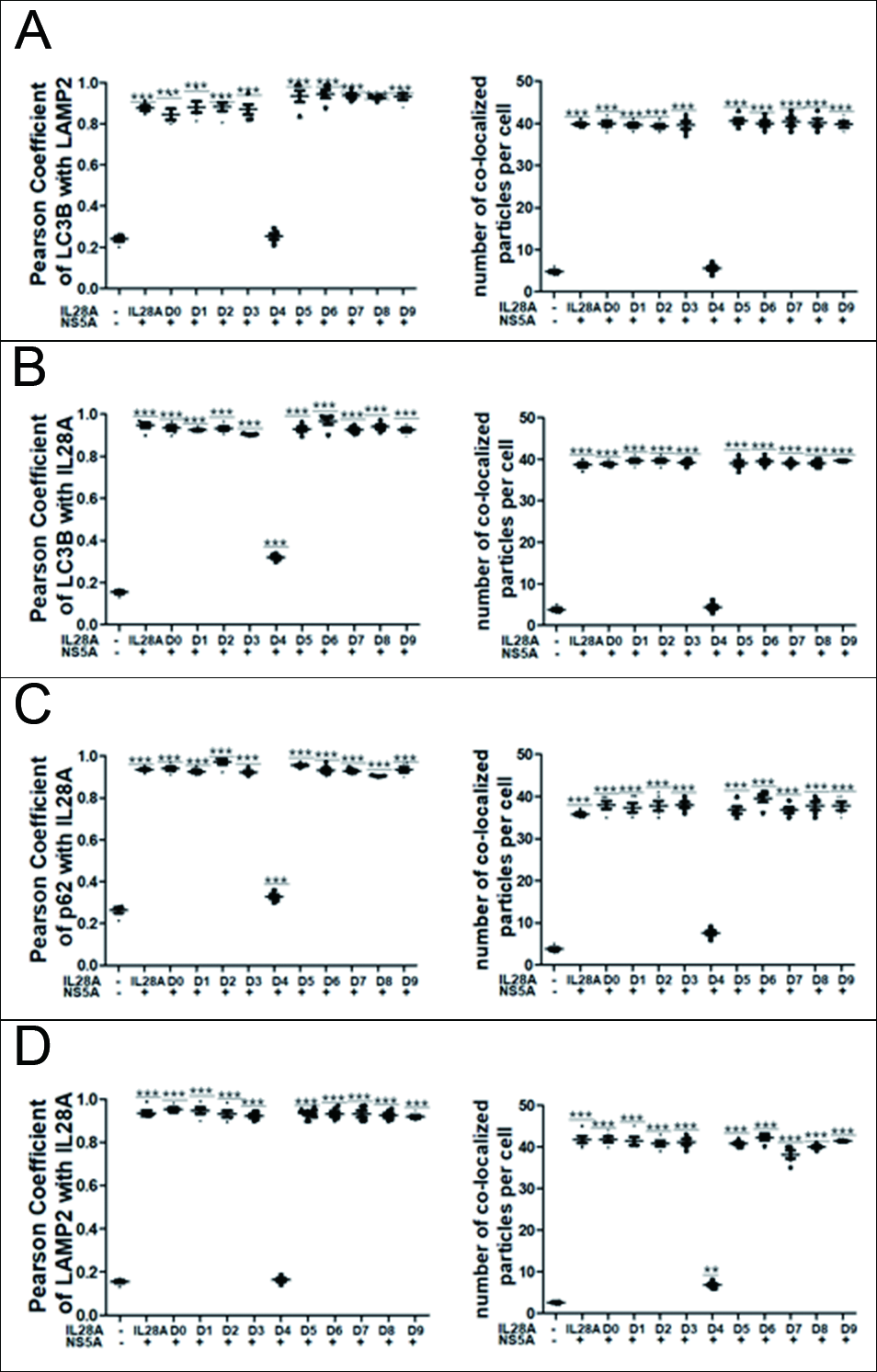

Supplement: Supplementary file 4 — Figure S3 [file 41419_2020_2400_MOESM4_ESM.tif]

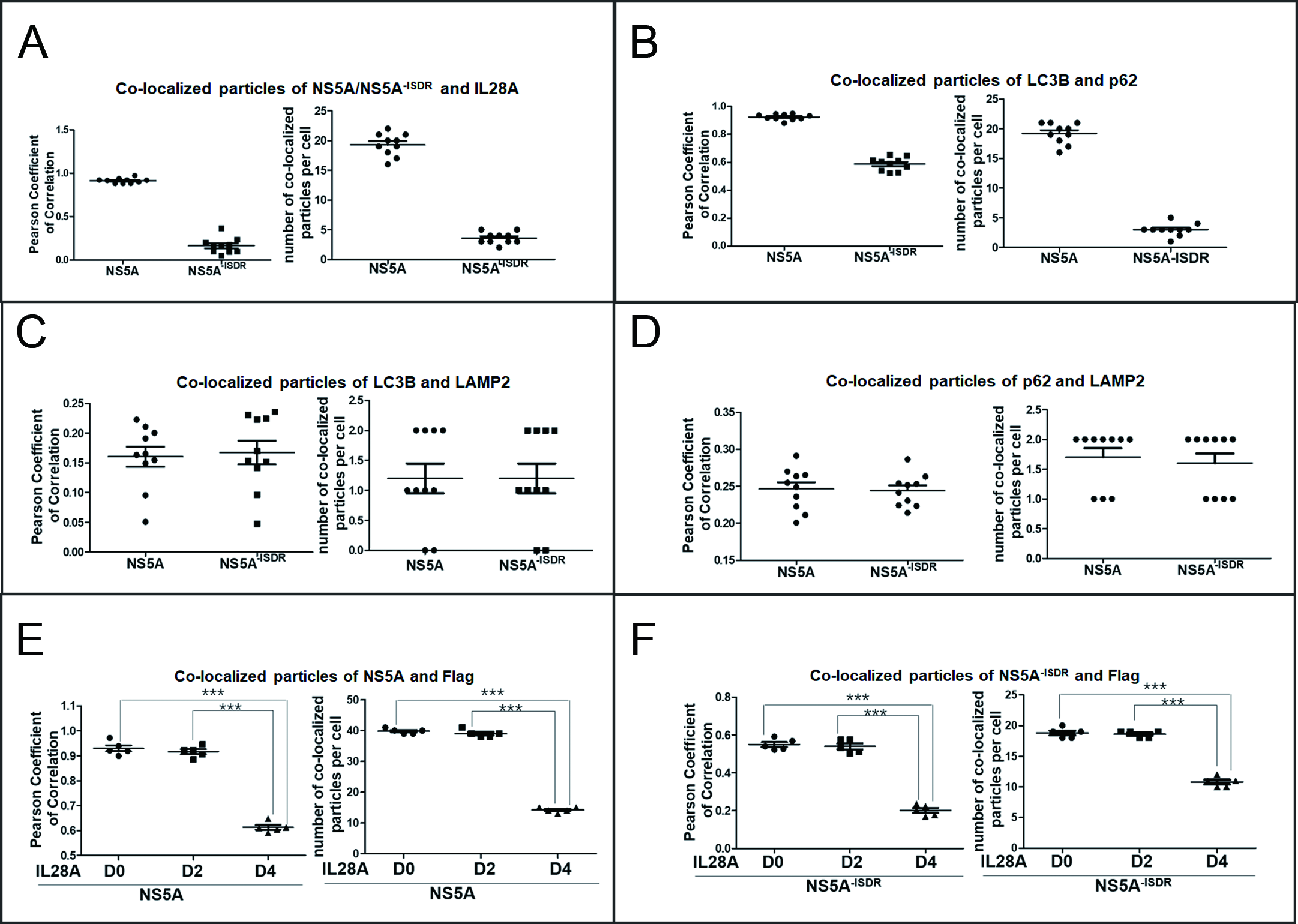

Supplement: Supplementary file 5 — Figure S4 [file 41419_2020_2400_MOESM5_ESM.tif]
